# Overexpression of MRP4 (ABCC4) and MRP5 (ABCC5) confer resistance to the nucleoside analogs cytarabine and troxacitabine, but not gemcitabine

**DOI:** 10.1186/2193-1801-3-732

**Published:** 2014-12-13

**Authors:** Auke D Adema, Karijn Floor, Kees Smid, Richard J Honeywell, George L Scheffer, Gerrit Jansen, Godefridus J Peters

**Affiliations:** Department of Medical Oncology, VU University Medical Center, PO Box 7057, 1007 MB Amsterdam, The Netherlands; Pathology, VU University Medical Center, PO Box 7057, 1007 MB Amsterdam, The Netherlands; Rheumatology, VU University Medical Center, PO Box 7057, 1007 MB Amsterdam, The Netherlands

**Keywords:** ABC pumps, MRP4 (ABCC4), MRP5 (ABCC5), Gemcitabine, Cytarabine, Troxacitabine

## Abstract

We aimed to determine whether the multidrug-resistance-proteins MRP4 (ABCC4) and MRP5 (ABCC5) confer resistance to the antimetabolites cytarabine (Ara-C), gemcitabine (GEM), and the L-nucleoside analog troxacitabine. For this purpose we used HEK293 and the transfected HEK/MRP4 (59-fold increased MRP4) or HEK/MRP5i (991-fold increased MRP5) as model systems and tested the cells for drug sensitivity using a proliferation test. Drug accumulation was performed by using radioactive Ara-C, and for GEM and troxacitabine with HPLC with tandem-MS or UV detection. At 4-hr exposure HEK/MRP4 cells were 2-4-fold resistant to troxacitabine, ara-C and 9-(2-phosphonylmethoxyethyl)adenine (PMEA), and HEK/MRP5i to ara-C and PMEA, but none to GEM. The inhibitors probenecid and indomethacin reversed resistance. After 4-hr exposure ara-C-nucleotides were 2-3-fold lower in MRP4/5 cells, in which they decreased more rapidly after washing with drug-free medium (DFM). Trocacitabine accumulation was similar in the 3 cell lines, but after the DFM period troxacitabine decreased 2-4-fold faster in MRP4/5 cells. Troxacitabine-nucleotides were about 25% lower in MRP4/5 cells and decreased rapidly in MRP4, but not in MRP5 cells. Accumulation of GEM-nucleotides was higher in the MRP4/5 cells. In conclusion: MRP4 and MRP5 overexpression confer resistance to troxacitabine and ara-C, but not to GEM, which was associated with a rapid decline of the ara-C and troxacitabine-nucleotides in HEK/MRP4-5 cells.

**Electronic supplementary material:**

The online version of this article (doi:10.1186/2193-1801-3-732) contains supplementary material, which is available to authorized users.

## Introduction

The ATP-binding cassette (ABC) transporters consist of a family of integral membrane proteins capable of unidirectional transport of a wide variety of compounds across cell membranes. Part of the natural function is protection against xenobiotics, by pumping them out of the cell. This transport by ABC transporters occurs against a concentration gradient made possible by ATP hydrolysis (Borst and Elferink^2002^). On the basis of sequence homology and domain organization the ABC family is subdivided into seven subfamilies (ABCA-ABCG) (Gottesman et al.^2002^). The ABCC (MRP) subfamily consists of nine related transporters (ABCC1-6, ABCC10-12 or MRP1-9); these MRP proteins have at least 2 hydrophobic transmembrane domains and 2 cytoplasmatic domains. The MRP family is subdivided according to the presence or absence of a third transmembrane domain; MRPs 1, 2, 3, 6 and 7 contain this third transmembrane domain, while the other MRPs don’t contain this domain. The presence of this third transmembrane domain is responsible for different substrate specificity between the MRPs possessing and lacking this domain (Borst and Elferink^2002^; Deeley et al^2006^; Gottesman et al.^2002^; Kruh and Belinsky^2003^).

Next to their natural function, most MRP transporters have been implicated in drug resistance, but they have a wide range of different substrate specificities (Deeley et al^2006^; Gottesman et al.^2002^; Kruh and Belinsky^2003^). However, their role in clinical drug resistance seems to be limited. The MRP transporters that don’t contain a third transmembrane domain, MRP4 (ABCC4), MRP5 (ABCC5) and MRP8 (ABCC11), are capable of transporting monophosphorylated nucleoside analogs, which can confer resistance to, amongst others, 6-mercaptopurine, 6-thioguanine and PMEA for MRPs 4 and 5 (Fukuda and Schuetz^2012^; Reid et al^2003^; Wijnholds et al^2000^), while MRP8 is also able to transport fluoropyrimidines (Kruh and Belinsky^2003^).

**Figure 1:**
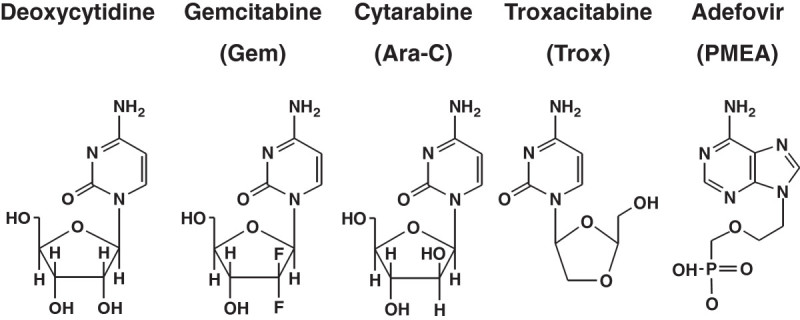
**Structural formulae of deoxycytidine, gemcitabine, cytarabine, troxacitabine and PMEA.**

Cytarabine and Gemcitabine are deoxynucleoside analogs frequently used in the treatment of solid (dFdC) (Heinemann^2002^; Hussain and James^2003^; Ramalingam and Belani^2008^) and hematological cancers (Ara-C) (Plunkett and Gandhi^1993^; Momparler^2013^). Troxacitabine is an experimental deoxynucleoside analog, which has an unnatural L-orientation; causing differences in uptake and metabolism compared to other deoxynucleoside analogs (Grove et al^1995^; Gourdeau et al^2001^; Gumina et al^2006^) (Figure 1). Resistance to deoxynucleoside analogs poses a limitation to the clinical efficacy in the treatment of cancer, making the treatment less effective and requiring higher doses with higher risks of side effects (Lage^2008^; Kruh^2003^). Limited information is available on the role of MRPs in drug resistance to nucleoside analogs. MRP4 and MRP5 have been implicated in resistance to thiopurines and phosphonates such as PMEA (Reid et al^2003^; Wielinga et al^2002^; Schuetz et al^1999^; Chen et al^2001^). MRP5 and MRP8 have also been shown to be involved in antimetabolite resistance (Pratt et al^2005^; Guo et al^2003^). The mechanism is based on efflux of the somewhat polar phosphonate and of the monophosphates of 6-mercaptopurine and 5-fluorouracil out of the cell; in contrast the more polar di- and triphosphates are not a substrate, similar to the efflux of methotrexate monoglutamate compared to the higher glutamate forms (Hooijberg et al^1999^; De Wolf et al^2008^). It was reported that for some nucleosides such as clofarabine and gemcitabine another ABC transporter (ABCG2) might confer resistance to a nucleoside analog, in which the nucleoside may be a substrate as well (De Wolf et al^2008^).

MRP 4 and 5 have related structures and show a similar ability to transport nucleotide analogs. However, gemcitabine is more effectively phosphorylated than Ara-C and troxacitabine, but troxacitabine shows a different uptake mechanism. Using HEK cells transfected with either MRP4 or MRP5, we investigated whether MRP4 and 5 were involved in the efflux of nucleoside analogs from cancer cells.

## Materials and methods

### Drugs

Cytarabine (Ara-C) was from Sigma-Aldrich (St. Louis, MO, USA), gemcitabine (dFdC) was from Eli-Lilly (Indianapolis, IN, USA), troxacitabine was from Shire BioChem (Laval, Quebec, Canada), PMEA was a gift from Prof. J. Balzarini, Rega Institute, Leuven, Belgium, tetrahydrouridine (THU) was from Calbiochem (Merck, Darmstadt, Germany), indomethacin and probenecid were from Sigma-Aldrich (St. Louis, MO, USA). ^3^H-cytarabine was obtained from Moravek (Brea, CA, USA). Rat monoclonal antibodies against MRP4 and MRP5 were used to detect the expression of MRP4 and MRP5, respectively (Wielinga et al^2003^; Lemos et al^2009^). As secondary antibody a horseradish peroxidase-conjugated rabbit-anti-rat antibody (DakoCytomation, Glostrup, Denmark) was used (Lemos et al^2009^).

### Cell lines

For the experiments the HEK human embryonic kidney cells transfected with MRP4 and MRP5i were used. The doubling times of the cell lines were 19, 17 and 16 hr, respectively. The cells were kindly provided by Dr. P.R. Wielinga from the Netherlands Cancer Institute (Wielinga et al^2003^). Earlier experiments did not show differences between the wild-type HEK and mock transfected cells. The cell lines were cultured in DMEM medium with glutamine (BioWhittaker, Verviers, Belgium), supplemented with 10% fetal calf serum (Gibco, New York, NY, USA) and 20 mM HEPES buffer (BioWhittaker).

Both HEK/MRP4 and HEK/MRP5i cells were characterized for the expression of MRP4 and MRP5, respectively, by performing a Western blot. These were performed as described earlier by Lemos et al (2009).

### Chemosensitivity assay

As chemosensitivity assay we used the SRB assay (Keepers et al^1991^) rather than a clonogenic assay. For the present purpose, differences in chemosensitivity, we earlier demonstrated that the SRB assay is an appropriate assay (Keepers et al^1991^; Pizao et al^1992^; Ferreira et al^2000^). Cells were transferred to 96 wells plates; on day one a serial dilution of one drug was added to the cells. The cells were incubated for 72 hours with drugs or for 4 hours with drugs and 68 hours with drug free medium. The relative amount of cells at drug addition was determined by fixing control wells at day 0 (drug addition) and processed as described below. The other (treated and control) cells were fixed after the incubation period, and washed and stained with SRB. The stained proteins were measured at 492 nm with an automated spectrophotometric microplate reader (Tecan, Salzburg, Austria); the measured optical density correlates with the amount of cells at the moment of fixing.

The data were plotted in a graph to give a growth inhibition curve, according to the guidelines as published by the NCI (http://dtp.nci.nih.gov/branches/btb/ivclsp.html). From this growth inhibition curve the IC_50_ value was determined by interpolating at the 50% growth level.

### Ara-C metabolism

Ara-C metabolism was investigated by a procedure described earlier using ^3^H-Ara-C (Adema et al^2012^). Shortly, cells were harvested and resuspended in fresh medium at 5x10^6^ cells/ml. Of this cell suspension 100 μl was used for each experiment. To inhibit deamination by CDA, THU was added at a final concentration of 100 μM (Yusa et al^1995^), the drugs were added to reach a final concentration of 8.7 μM for Ara-C (specific activity 1789 mCi/mmol). The cells were incubated for 4 hours at 37°C. In order to measure drug retention the drug-containing medium was replaced after 4 hours and the cells were incubated for 2 hours in drug free medium. Thereafter the cells were spun down (3000 g, 2 min, 4°C) and the medium was stored as extracellular fraction at -20°C. The cells were washed with cold PBS (12000 g, 1 min, 4°C). The cell pellet was resuspended in 45 μl cold PBS and extracted by addition of 5 μl perchloric acid (5 M) and chilled on ice for 20 minutes. The supernatant containing the cytosolic fraction was neutralized with 10 μl KH_2_PO_4_ (5 M) and stored as intracellular fraction at -20°C.

Of the extracellular and intracellular cytosolic samples 5 μl was spotted onto a plastic backed silica thin-layer chromatography plate (Merck KgaA, Darmstadt, Germany). The chromatography was performed with chloroform/methanol (3:2 v/v) as a mobile phase. After separation the spots were visualized with UV light and cut into separate scintillation vials, and radioactivity was eluted by overnight incubation in methanol. The samples were measured together with the perchloric acid pellet samples in an LSC counter and quantified as disintegrations per minute (dpm).

### Accumulation of dFdCTP

Cells were treated with 25 μM of dFdC. The cells were incubated for 4 hours and retention of the triphosphates was investigated after 2 and 4 hours incubation in drug-free medium. Thereafter the cell pellet was resuspended in 150 μl ice-cold PBS and incubated for 20 minutes at 4°C with 40% trichloroacetic acid. After centrifugation (10,000 g, 10 min, 4°C) the supernatant was treated with a 2-fold excess of trioctylamine/1,1,2-trichlorotrifluorethane (1:4) and spun down (10,000 g, 1 min) and the aqueous phase was stored at -20°C. For measurement of gemcitabine triphosphate (dFdCTP) these samples were analyzed by HPLC on a Whatman Partisphere SAX column (GE healthcare, Chalfont St. Giles, UK) using isocratic gradient elution as described earlier (Ruiz van Haperen et al^1994^; Noordhuis et al^1996^). The accumulation of dFdCTP was expressed relative to the concentration of ATP.

### Accumulation of Troxacitabine and its phosphates

Cells were treated with 10 μM troxacitabine and were extracted similarly as described for dFdCTP. An aliquot of the cell pellet was retained for protein determination. For measurement of troxacitabine and its nucleotides, the acid-precipitated aliquots of troxacitabine treated samples were split, one for troxacitabine and the other for the nucleotides. The latter aliquot was incubated with ammonium bicarbonate and alkaline phosphatase at 37°C for 2 hours. Thereafter the samples were cleaned of alkaline phosphatase by the addition of ice-cold isopropyl alcohol. The supernatant was cleaned of inorganic content by freeze drying and reconstituting in ethyl acetate. After back extraction with water the aqueous layer was used for LC-MS/MS analysis. Mass spectroscopic sensitivity and analytical parameters of troxacitabine were established as the molecular ion [M + H] + of 214.2 and a product ion of 112 amu corresponding to the nucleoside base, which is a typical fragmentation pattern for nucleosides and nucleoside analogues (Honeywell et al^2007^). Analysis of troxacitabine and its nucleotides was performed similarly to that described earlier for deoxynucleoside analogs (Sigmond et al^2009^; Bijnsdorp et al^2011^). Chromatography consisted of a gradient system using 100% aqueous formic acid (0.1%) as buffer A and 40% aqueous formic acid (0.1%)/60% methanol for buffer B. The gradient consisted of an initial 2 minute hold at 96% buffer A followed by an increase to 100% buffer B over 10 minutes with a Phenomenex 100 × 2.0 mm ODS3, 3 μm column maintained at 30°C. Troxacitabine eluted at 1.20 minutes. LC-MS/MS determines the concentration in ng/ml of the aliquot taken. After taking dilution factors into consideration, the number of moles of troxacitabine is determined for each cell pellet before and after incubation with alkaline phosphatase. The first measurement is the free troxacitabine and the difference between the two measurements is the total phosphorylation product of troxacitabine. Each result is adjusted for the either the protein content of the cell pellet or the cell count of the original pellet.

### Statistics

Significance of differences between wild-type and MRP4 or MRP5 transfected cells, and of different treatments, was evaluated with Student’s t-test using the options provided in either the Excel or Prism Graphpad program. Depending on the research question, testing was paired or unpaired. Cut-off for significance was set at p < 0.05.

## Results

**Figure 2:**
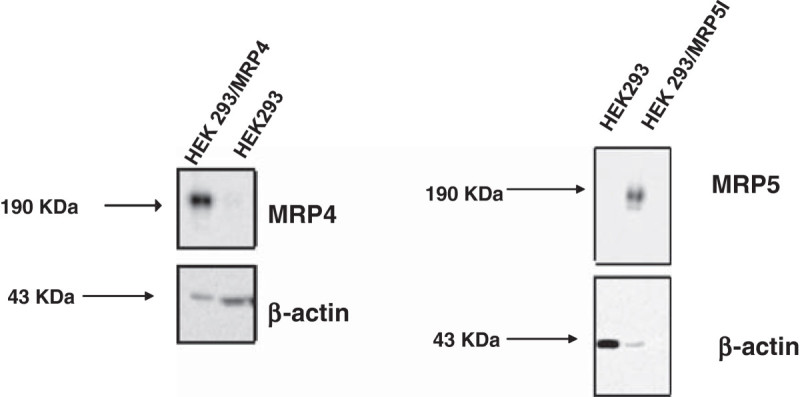
**Expression of MRP4 and MRP5 in the parent HEK cells and the resistant variants HEK/MRP4 and HEK/MRP5i.** For MRP5i cells loading of proteins had to be decreased to prevent overloading.

Transfected HEK cells were characterized by Western blotting by using specific antibodies against MRP4 and MRP5 (Lemos et al^2009^). Both variants had a high overexpression of either MRP4 or MRP5. Due to the transient nature of transfections, the cells had to be challenged regularly and were checked for their expression (Figure 2). However, using this procedure expression of MRP4 and MRP5 in these cell lines was stable throughout the course of each experiment and similarly high between experiments. Earlier we also demonstrated that expression of nucleoside activating enzymes such as deoxycytidine kinase does not change during this period (data not shown).

**Figure 3:**
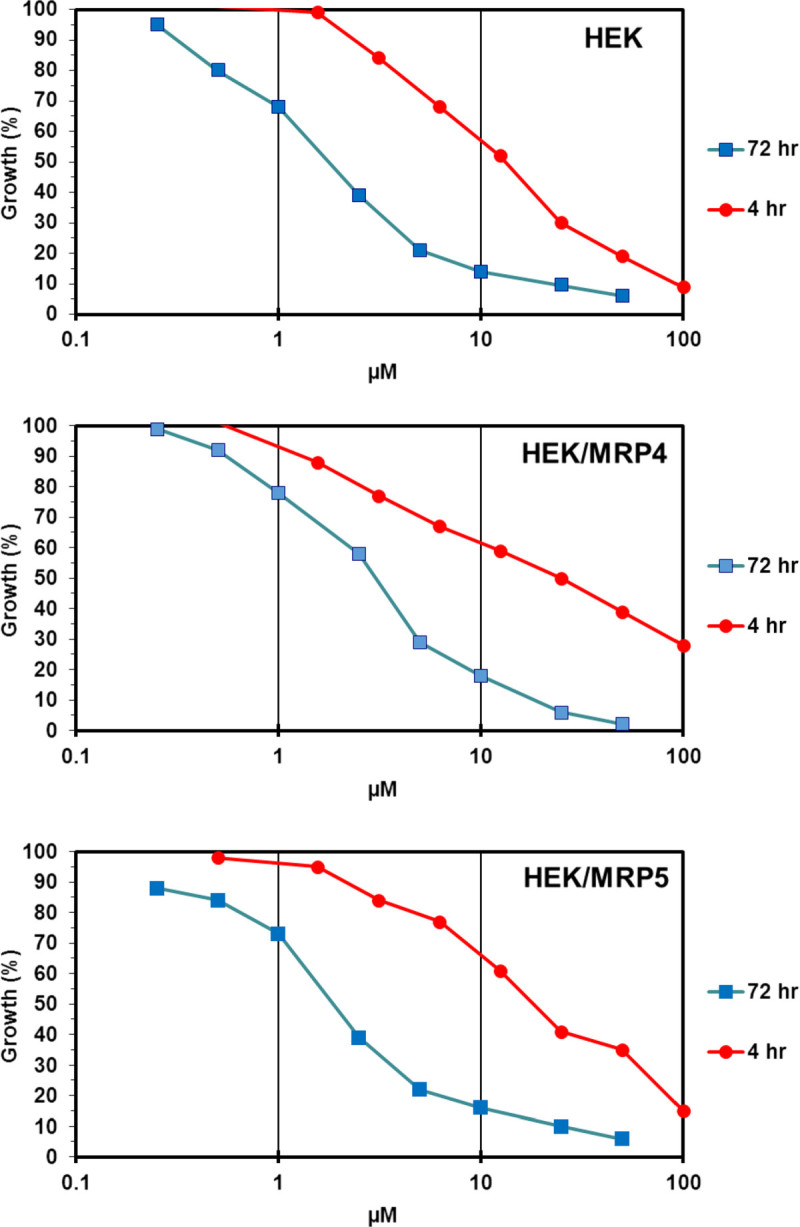
**Representative growth curves for troxacitabine showing the different sensitivity at long (72 hr) and short (4 hr) term exposure.**

HEK cells, wild-type and transfected variants, were exposed to the drugs for 4 and 72 hours (Figure 3). Previously we have shown for several deoxynucleoside analogs that cells were less sensitive at a short exposure (Ruiz van Haperen et al^1994^). Although cross-membrane transport of nucleoside analogs is rapid, their intracellular accumulation is much slower and depends on characteristics of each cell line and is different for each nucleoside analog (Adema et al^2012^; Yusa et al^1995^; Ruiz van Haperen et al^1994^; Plunkett and Gandhi^1993^). Hence, on the short term (less than 4 hr) this different metabolism leads to a low accumulation of Ara-C triphosphate and a much higher accumulation of gemcitabine-triphosphate; for Ara-C at 4 hr this also results in a relatively large proportion of monophosphates (Ruiz van Haperen et al^1994^; Noordhuis et al^1996^). These are relative good substrates for efflux pumps in contrast to the triphosphates (Reid et al^2003^; Wielinga et al^2002^; Pratt et al^2005^). Therefore we reasoned that a role for MRP4 and MRP5 would be more pronounced at a short incubation as was also found for antifolates (Hooijberg et al^1999^). The 4/72 hr ratio of IC50 values was higher in MRP4 and MRP5 cells compared to the wild type for troxacitabine (5.9 and 7.1 vs 3.3) and Ara-C (4.3 and 4.4 vs 3.3), but not for dFdC (Table 1).

**Table 1:** **Sensitivity of HEK cells to troxacitabine, Ara-C and gemcitabine in comparison to PMEA**

	Time	HEK	HEK/MRP4	RF	HEK/MRP5i	RF
Troxacitabine	4 h	11.0 ± 2.6	29.0 ± 4.0**	2.6	14.2 ± 3.0^**+**^	1.3
	72 h	3.3 ± 0.2	5.0 ± 0.8	1.5	2.0 ± 0.1^**+**^	0.61
Ratio IC50	4/72 h	3.3	5.9		7.1	
Ara-C	4 h	8.3 ± 0.6	23.3 ± 4.4*	2.8	17.7 ± 1.5**	2.1
	72 h	2.5 ± 1.5	5.4 ± 1.6	2.2	4.0 ± 1.5	1.6
Ratio IC50	4/72 h	3.3	4.3		4.4	
Gemcitabine	4 h	0.43 ± 0.08	0.60 ± 0.12	1.4	0.50 ± 0.06	1.2
	72 h	0.046 ± 0.006	0.061 ± 0.008	1.3	0.07 ± 0.01	1.5
Ratio IC50	4/72 h	9.3	10.0		7.1	
PMEA	4 h	600 ± 40	2500 ± 100**	4.2	2567 ± 550**	4.3

Value (in μM) are means ± SE of 3-4 separate experiments.

Statistical difference: significantly different from wild-type cells (^**+**^,P < 0.05; *,P<0.02; **,P < 0.01).

HEK/MRP5i more sensitive than HEK/MRP4: ^**+**^,P < 0.05. RF, resistance factor, ratio between IC50 of MRP4 or MRP5 cells divided by that of HEK wild type cells.

Exposure of the MRP 4 and 5 overexpressing cell lines to Ara-C, dFdC, troxacitabine and PMEA showed a differential resistance compared to the parental cell line both at 4 compared to 72 hours exposure (Table 1). PMEA was used as a positive control since this compound is known to be a substrate for MRP4 and MRP5 (Reid et al^2003^). The modest 4-fold resistance was within the expected range. However, the highest resistance, although still modest, was observed for troxacitabine in the HEK/MRP4 cell line, while no real resistance was observed in the HEK/MRP5 cell line. Resistance to Ara-C was observed in both the overexpressing cell lines. The effects at 72 hours exposure were either absent (dFdC) or lower. No resistance was seen for dFdC; for troxacitabine just a low1.4-fold in MRP4 cells and for Ara-C an equally low 2- and 1.6-fold in MRP4 and MRP5 cells, respectively.

**Figure 4:**
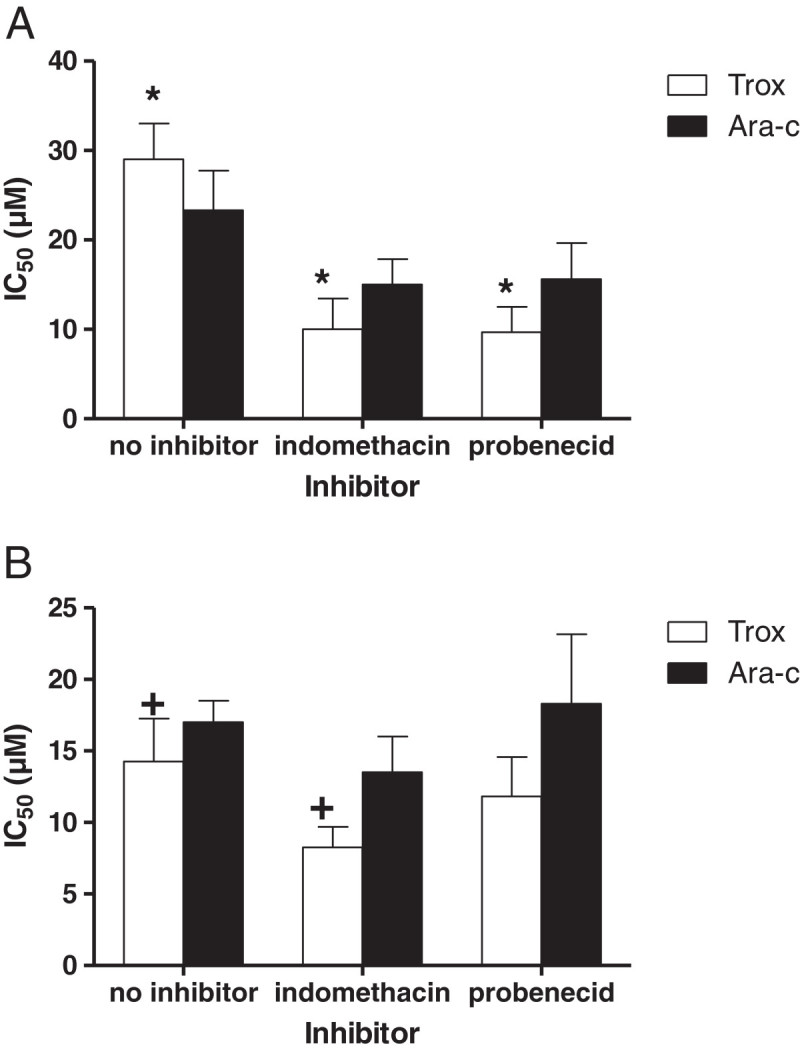
**Effect of the MRP4 and MRP5 inhibitors on sensitivity to troxacitabine (trox) and Ara-C in the HEK/MRP4 (a) and HEK/MRP5i (b) cell lines.** Values represent IC_50_ (μM) ± SEM after 4 hours exposure to drugs and inhibitor of at least 3 separate experiments. Thereafter cells were cultured in drug-free medium and drug sensitivity was measured 68 hr thereafter with the SRB assay. The effect of indomethacin and probenecid on troxacitabine sensitivity was significant compared to the control (no inhibitor) in HEK/MRP4 cells (*,p < 0.01); in HEK/MRP5i cells only the effect of indomethacin was significant (+,p < 0.05) (paired t-test)

For both Ara-C and troxacitabine we investigated whether inhibition of MRP4 or MRP5 would increase sensitivity. The inhibitors indomethacin and probenecid increased sensitivity to both Ara-C and troxacitabine in HEK/MRP4 cells (Figure 4a); inhibition of MRP5 with indomethacin only increased the sensitivity to Ara-C (Figure 4b). Although these transporters are not specific, they would only inhibit MRP4 or MRP5, since that is the only detectable transporter in the transfected cells. Moreover, in wild-type cells neither indomethacin or probenecid affected the sensitivity of either drugs, excluding the possibility that inhibition of another transporter might be responsible for the observed effect in the transfected cells.

**Figure 5:**
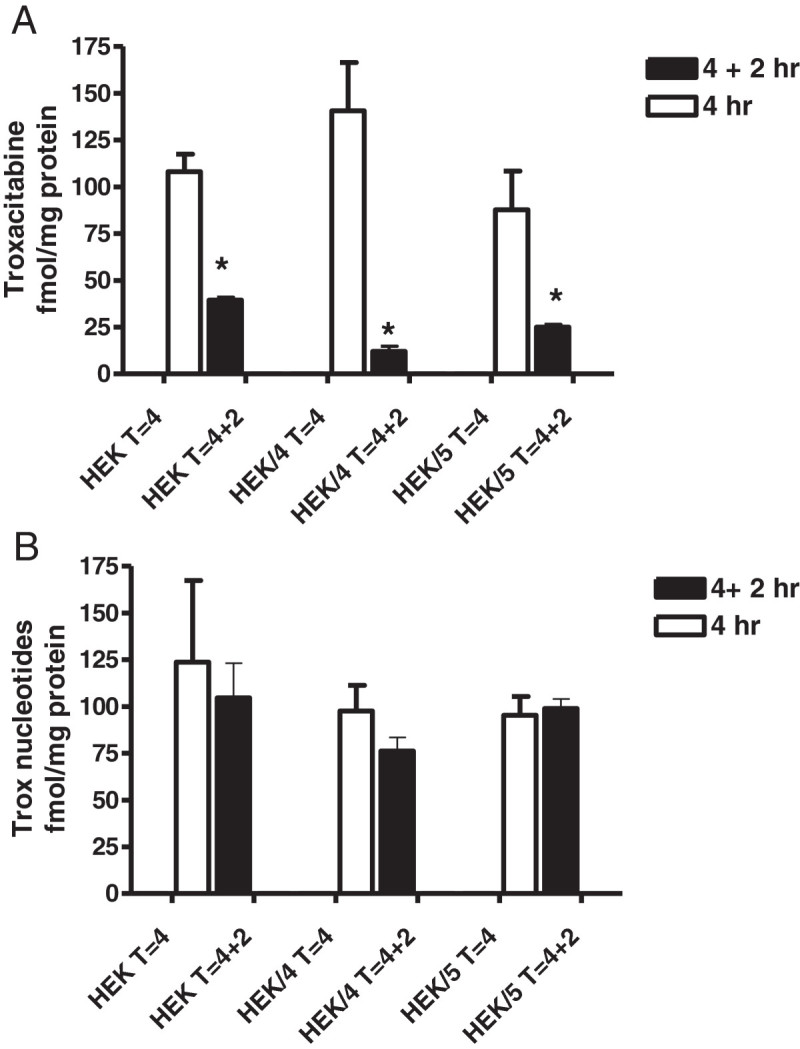
**Accumulation and retention of free troxacitabine (trox) (a) and troxacitabine-nucleotides (b) in the HEK, HEK/MRP4 (HEK/4) and HEK/MRP5i (HEK/5) cell lines.** T = 4: 4 hours exposure to 10 μM troxacitabine, T = 4 + 2: 4 hours exposure to troxacitabine followed by 2 hours drug-free medium. Values represent fmol/μg protein ± SEM of 3 separate experiments. For free troxacitabine the effect of drug-free medium was significant (*,p < 0.01) (paired t test). Accumulation was measured in the absence of indomethacin or probenecid.

To further elucidate the mechanisms explaining the resistance we investigated the accumulation and retention of troxacitabine, Ara-C, dFdC and their respective phosphorylated forms. Accumulation of phosphorylated troxacitabine was lower in both the HEK/MRP4 and HEK/MRP5i cell lines compared to the wild-type HEK cells (Figure 5a), this decrease was most pronounced in the MRP4 cell line after 4 hours exposure to troxacitabine and after 2 hours incubation in drug-free medium. Next to the phosphorylated troxacitabine, the free troxacitabine also showed a markedly decreased retention in both the HEK/MRP4 and the HEK/MRP5i cell lines (Figure 5b), which was most pronounced in the HEK/MRP4 cell line. None of the nucleotides was detectable outside the cells; apparently in case they are effluxed their concentration would be so far diluted that it would be even below the detection limit of our sensitive LC-MS-MS assay (troxacitabine) or radioactivity (ara-C).

**Figure 6:**
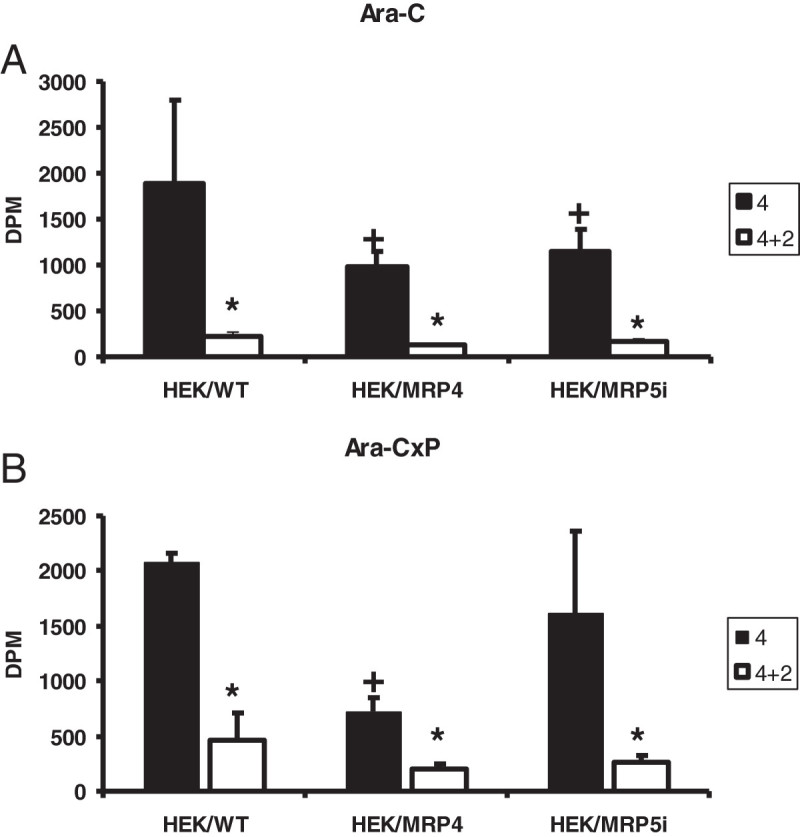
**Accumulation and retention of free Ara-C (a) and phosphorylated Ara-C (Ara-CxP) (b) in the HEK, HEK/MRP4 and HEK/MRP5i cell lines.** T = 4: 4 hours exposure to 10 μM Ara-C, T = 4 + 2: 4 hours exposure to Ara-C followed by 2 hours drug-free medium. Values represent DPM ± SEM of 3 separate experiments. For both free Ara-C and Ara-CxP the effect of drug-free medium was significant (*, p < 0.01) (paired t test). For free Ara-C the accumulation was significantly lower in HEK/MRP4 and HEK/MRP5i cells compared to wild-type HEK cells (+, p < 0.05) (unpaired t-test), for the Ara-CxP this only holds for HEK/MRP4 cells. Accumulation was measured in the absence of indomethacin or probenecid.

Accumulation of both Ara-C and phosphorylated Ara-C were lower in both the HEK/MRP4 and HEK/MRP5i cell lines compared to the wild-type HEK cells (Figure 6), however the decrease was most pronounced in the HEK/MRP4. The differences in both Ara-C and phosphorylated Ara-C were larger after incubation in drug-free medium. In all three cell lines the concentration of Ara-C decreased about 10-fold during incubation in drug-free medium, but that of Ara-C nucleotide only 5-fold in the wild-type. However, in both MRP4 and MRP5 cells remaining nucleotides were much lower than in the wild-type cells.

**Figure 7:**
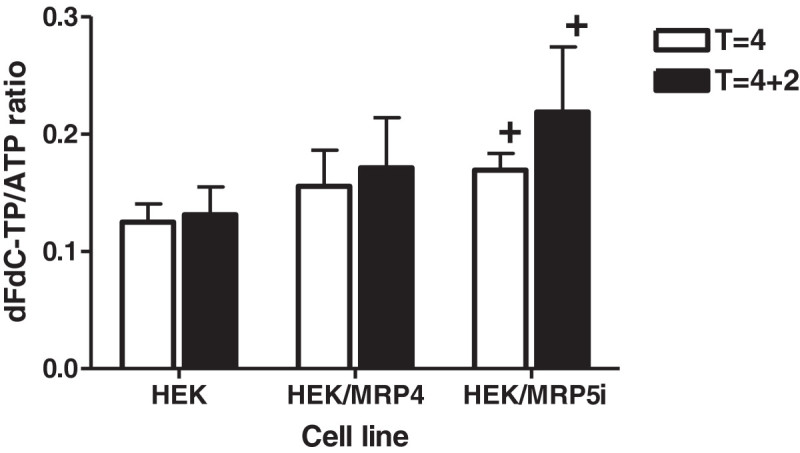
**Accumulation and retention of dFdCTP in the HEK, HEK/MRP4 and HEK/MRP5i cell lines.** T = 4: 4 hours exposure to 25 μM dFdC, T = 4 + 2: 4 hours exposure to dFdC followed by 2 hours drug-free medium. Values represent dFdCTP/ATP ratio ± SEM of 3 separate experiments. The accumulation of dFdCTP was significantly higher in HEK/MRP5i cells compared to wild type HEK cells (+, p < 0.05) (unpaired t test). Accumulation was measured in the absence of indomethacin or probenecid.

The accumulation and retention of dFdCTP showed a completely different pattern compared to Ara-C and troxacitabine. First, after 4 hours exposure dFdCTP accumulation was higher in the MRP4 and MRP5 cells compared to the wild-type HEK (Figure 7). Furthermore dFdCTP accumulation continued to increase upon withdrawal of gemcitabine both after exposure to 25 or 50 μM gemcitabine (data not shown).

## Discussion

In this study we have shown that overexpression of MRP4 or 5 confers resistance to the anti-cancer nucleoside analog cytarabine, due to efflux of Ara-CMP mediated by MRPs 4 or 5. The L-orientated nucleoside analog troxacitabine was a substrate for both MRP4 and MRP5, as well as its mono-phosphate appeared to be. On the other hand no evidence was obtained that the nucleoside analog gemcitabine or its nucleotides are a substrate for either MRP 4 or 5.

Most MRP transporters have been implicated in cellular drug resistance, but their potential role in clinical drug resistance has still not been clarified. They have a wide range of substrate specificities (Deeley et al^2006^; Kruh and Belinsky^2003^). It was previously shown that various anti-viral mono-phosphorylated nucleoside analogs are substrates to MRPs that don’t contain a third transmembrane domain (Reid et al^2003^; Wielinga et al^2002^; Schuetz et al^1999^; Chen et al^2001^; Pratt et al^2005^; Guo et al^2003^). The MRPs that don’t contain this third transmembrane domain are MRP 4, 5 and 8. As a positive control we included PMEA, which indeed showed a resistance in the MRP4 and MRP5 cells, similar to that published earlier (Reid et al^2003^; Fukuda and Schuetz^2012^). Also the lack of a difference for ara-C and gemcitabine at a long exposure (72 hr) was shown earlier (Reid et al^2003^), but shorter exposures were not tested. Resistance to Ara-C and troxacitabine were in the same range, indicating a similar mechanism of drug efflux.

The data show that Ara-CMP, troxacitabine monophosphate and troxacitabine are substrates for MRP4. The sensitivity experiments showed the largest extent of resistance for these analogs in the MRP4 transfected cell line, while this resistance was reversible by the inhibition of MRP4. The reduction in accumulation and retention of both the unphosphorylated and phosphorylated analogs underlined these results. Cytarabine monophosphate may also be a substrate for MRP5, indicated by the decreased sensitivity in the transfected cell line, the decrease in accumulation and retention of both the unphosphorylated and phosphorylated cytarabine.

Literature data on resistance to deoxynucleoside analogs due to increased efflux is scarce. It was shown that ABCG2 (BCRP) might confer resistance to the deoxynucleoside analogs clofarabine and gemcitabine (De Wolf et al^2008^), while MRP5 and MRP8 may confer resistance to fluoropyrimidines by effluxing the mononucleotides (Kruh and Belinsky^2003^; Pratt et al^2005^; Guo et al^2003^; Li et al^2011^). A similar pattern was found for methotrexate, for which a resistance was found for MRP1-5 and BCRP (Hooijberg et al^1999^; De Wolf et al^2008^; Wielinga et al^2005^). A common denominator for all these resistance patterns was the fact that all compounds need to be activated to a polar metabolite, which appeared to a substrate for these efflux pumps. However, usually this substrate specificity was limited to the somewhat polar phosphonate (PMEA), the mononucleotide (Clofarabine, 5-fluorouracil, 6-mercaptopurine) or the monoglutamate, the least polar metabolite from these metabolite drugs. Since the di- and triphosphate forms of nucleotides and the polyglutamates derivatives of methotrexate (e.g. di to penta glutamates), are much more polar they are poor substrates for the MRP4 and MRP5 pumps. Only BCRP was able to efflux some of the polyglutamates. Another common denominater was that resistance was usually only found at short exposure to the drugs, especially with drugs which have a rapid metabolism to the nucleotides or polyglutamates, such as gemcitabine and tomudex, respectively (Hooijberg et al^1999^). Hence, only for troxacitabine and ara-C a resistance was found, since these drugs are not metabolized rapidly enough to form a triphosphate after 4 hr. Another novel finding of our data is that troxacitabine is also a substrate for MRP4, in contrast to unphosphorylated Cytarabine or gemcitabine. This is possibly because troxacitabine is more polar then cytarabine (Gourdeau et al^2001^); another difference of the L-nucleoside troxacitabine compared to other non-L nucleoside analogs, is that is not a substrate for the human equilibrative nucleoside transporter (Gourdeau et al^2001^). Another interesting finding of our data is that gemcitabine is not a substrate for MRP4 or MRP5, probably because it is converted to gemcitabine triphosphate too rapidly to be effluxed (Fukuda and Schuetz^2012^). This is confirmed by the sensitivity, which is only marginally decreased in the transfected cell lines. It can however, not be excluded, that under ideal conditions, such as poor metabolism to more polar nucleotides, gemcitabine monophosphate might be a substrate. However, the transfected HEK cells are already relatively poor gemcitabine metabolizers compared to other cell lines tested in our laboratory for gemcitabine (Ruiz van Haperen et al^1994^; Noordhuis et al^1996^). Although the mechanisms were not elucidated some evidence was presented that gemcitabine and Ara-C resistance might be related to MRP7 overexpression (Ikeda et al^2011^; Hopper-Borge et al^2009^). For Ara-C it was shown that this was due to an increased efflux of Ara-CMP (Hopper-Borge et al^2009^).

Since MRP4 and MRP5 are relatively wide distributed (Hooijberg et al^2006^; Fukuda and Schuetz^2012^), our findings may have consequences for the application of these drugs in the clinic, i.e. how can a potential resistance mechanism be bypassed? In this paper we focused on the role of MRP4 and MRP5 using two syngenic HEK cell lines, instead of using cell lines with intrinsic or acquired resistance to either ara-C, troxacitabine or gemcitabine. In the latter models one might have to deal with other mechanisms, while transfected, syngeneic cell lines are clean in this aspect. Of course other mechanisms in the complex setting of a patient, cannot be excluded. These include among others, limited transport, decreased phosphorylation and alterations in the target (Plunkett and Gandhi^1993^). For nucleoside analogs such as Ara-C and gemcitabine it has been demonstrated earlier (Plunkett & Gandhi^1993^; Momparler^2013^; Ruiz van Haperen et al^1994^; Noordhuis et al^1996^) that their effect is schedule dependent; at long incubation their sensitivity is increased compared to a short exposure. This is due to a relatively slow formation of the active nucleotides. Therefore administration schedules for Ara-C are relatively long (Momparler^2013^); and it has been proposed that gemcitabine should also be given in a longer infusion that the standard 30 min infusion. Indeed a 24-hr infusion with gemcitabine showed the best antitumor effect in mice (Veerman et al^1996^). Plunkett et al suggested giving gemcitabine in a longer infusion to optimize gemcitabine phosphorylation; the so-called fixed rate infusion of 10 mg/min/m^2^ seemed to give a better effect in a phase 2 study, although the results were not confirmed in a randomized phase 3 (Tempero et al^2003^; Poplin et al^2009^). In case gemcitabine would be a substrate for not only MRP7 but also other ABC transporters, such a schedule would serve two purposes, a prolonged period of gemcitabine exposure would increase gemcitabine phosphorylation, while the length of exposure would also prevent efflux of gemcitabine-MP. Alternatively, a similar effect was observed with a gemcitabine prodrug, CP-4126 (now CO1.01), which showed a prolonged retention in the cells, resembling a long exposure (Adema et al^2012^).

In this paper we focused on the use of specific model systems (wild type cells transfected MRP4 or MRP5) instead of cancer cell lines with a naturally high expression of any of the MRPs, including MRP4, 5 or 8. In cancer cells another potential resistance mechanism would easily overrule a MRP mediated resistance, which did not happen in our models. Also the use of primary cancer cells would give the same problem, since they are almost by definition characterized by resistance due to multiple alterations in the cells. Hence a resistance to 6-mercaptopurine due to MRP4 overexpression (Peng et al^2008^) or to cyclic nucleotides in cells characterized by MRP8 overexpression (Guo et al^2009^), may have a similar denominator (a polar nucleotide which cannot efflux out of the cell), but does not explain the differences found between e.g. ara-C and gemcitabine, which is based on metabolism, an aspect not covered earlier in literature.

In conclusion, we have demonstrated that the ABC transporters MRP4 and MRP5 may play a role in nucleoside resistance by effluxing the monophosphate. This resistance may be bypassed by prolonged exposure.

## Authors’ original submitted files for images

Below are the links to the authors’ original submitted files for images. Authors’ original file for figure 1 Authors’ original file for figure 2 Authors’ original file for figure 3 Authors’ original file for figure 4 Authors’ original file for figure 5 Authors’ original file for figure 6 Authors’ original file for figure 7

## Abbreviations

ABC transporters: ATP-binding cassette transporters
Ara-C: Arabinosyl-cytosine-cytarabine
Ara-CMP: Cytararabine monophosphate
CDA: Cytidine deaminase
dFdC: Gemcitabine, 2′,2′-difluoro-2′-deoxycytidine
dFdCTP: Gemcitabine triphosphate
DMEM: Dulbecco’s Modified Eagle Medium
MRP: Multidrug resistance associated protein
PMEA: 9-(2-phosphonylmethoxyethyl)adenine
PBS: Phosphate buffered saline
SRB: Sulforhodamine-B
THU: Tetrahydrouridine
TROX: Troxacitabine.
