# Supplementary figures and images for: Overexpression of MRP4 (ABCC4) and MRP5 (ABCC5) confer resistance to the nucleoside analogs cytarabine and troxacitabine, but not gemcitabine
Source: Springerplus. 2014 Dec 13;3:732. doi: 10.1186/2193-1801-3-732 (PMC4320143; doi:10.1186/2193-1801-3-732)

**Deoxycytidine**

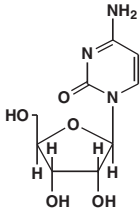

**Gemcitabine**  
(Gem)

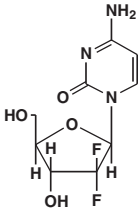

**Cytarabine**  
(Ara-C)

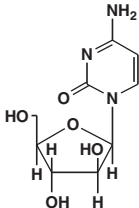

**Troxacitabine**  
(Trox)

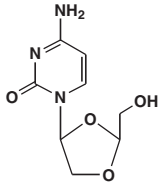

**Adefovir**  
(PMEA)

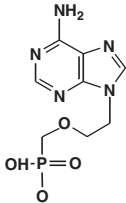

Supplement: Supplementary file 1 — Authors’ original file for figure 1 [file 40064_2014_1514_MOESM1_ESM.pdf]

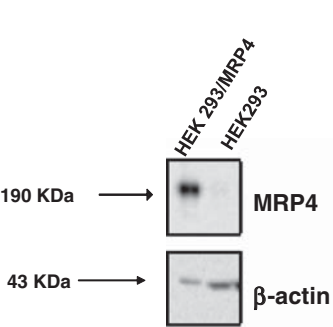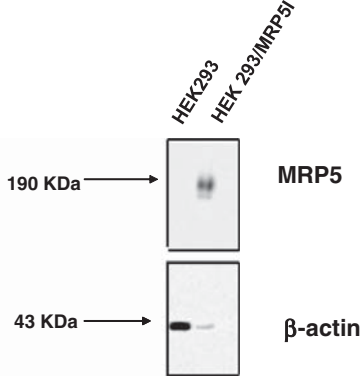

Supplement: Supplementary file 2 — Authors’ original file for figure 2 [file 40064_2014_1514_MOESM2_ESM.pdf]

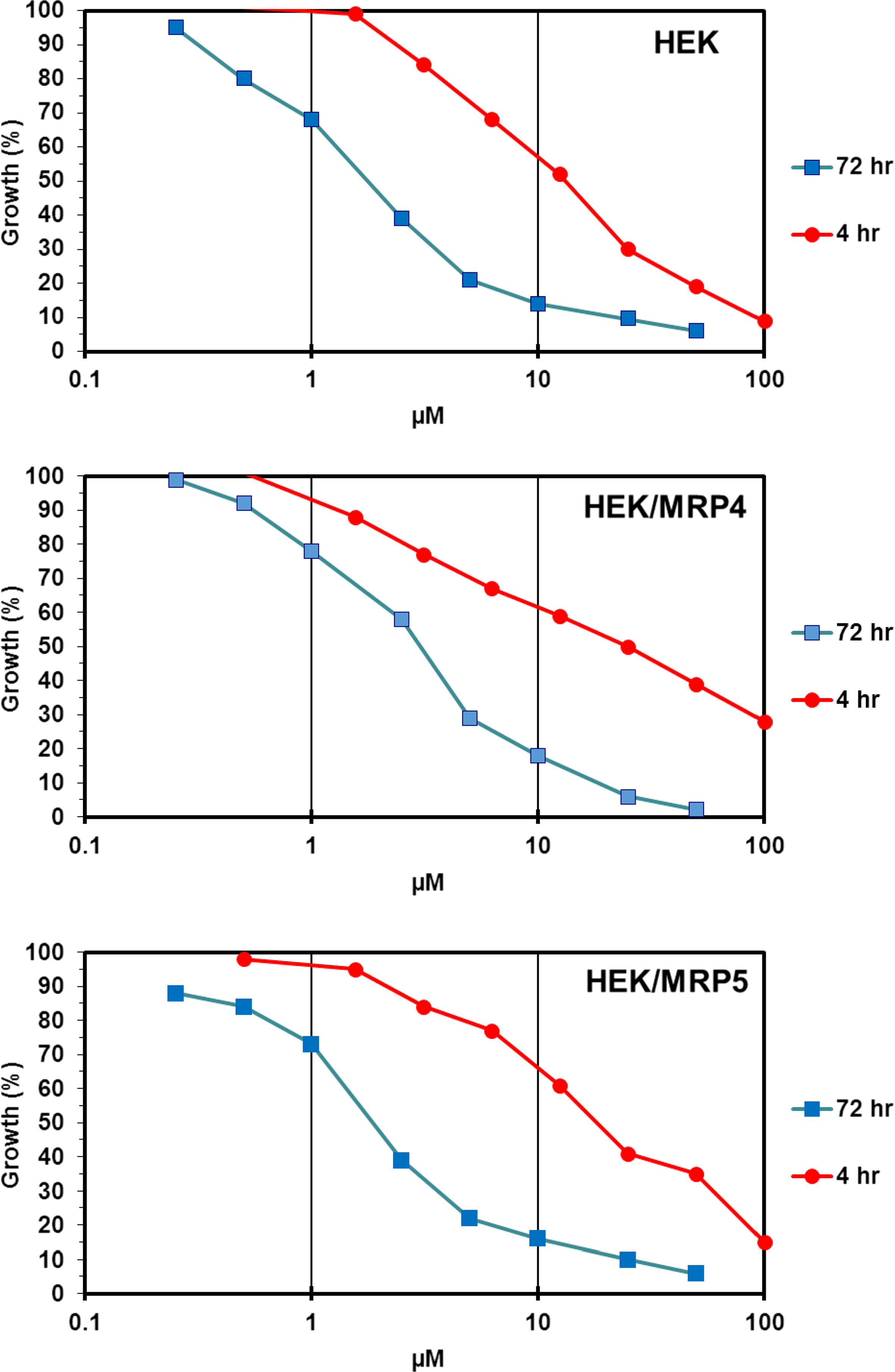

Supplement: Supplementary file 3 — Authors’ original file for figure 3 [file 40064_2014_1514_MOESM3_ESM.tif]

**A**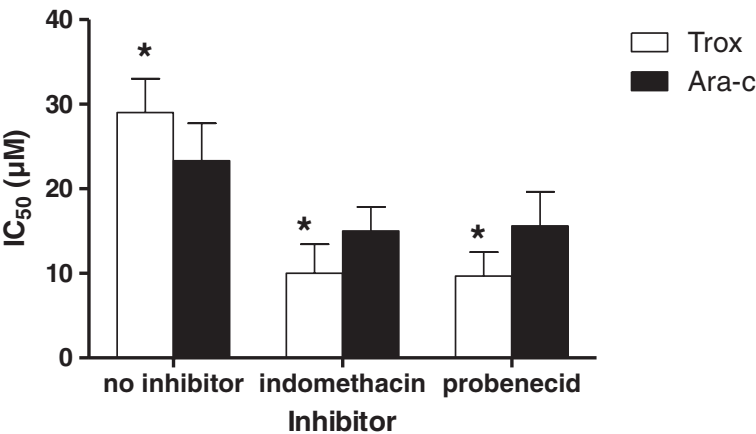**B**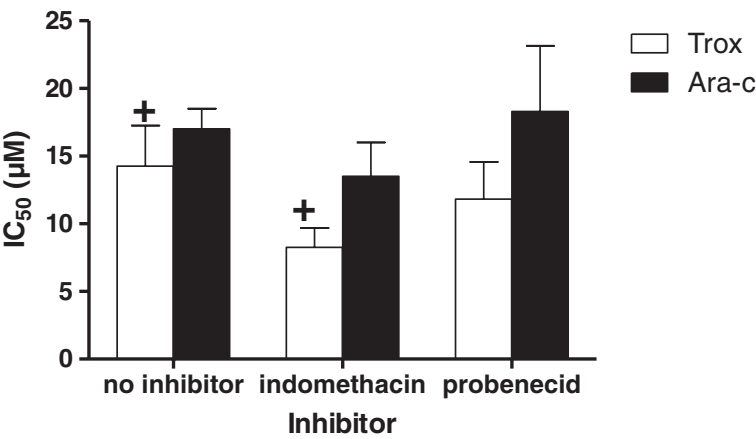

Supplement: Supplementary file 4 — Authors’ original file for figure 4 [file 40064_2014_1514_MOESM4_ESM.pdf]

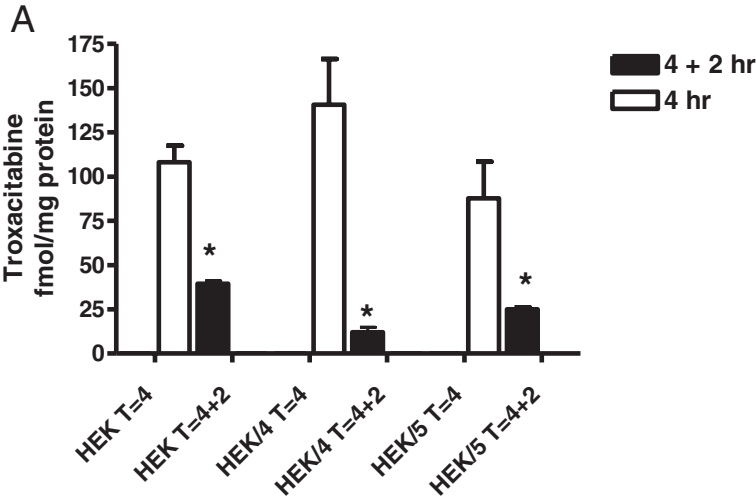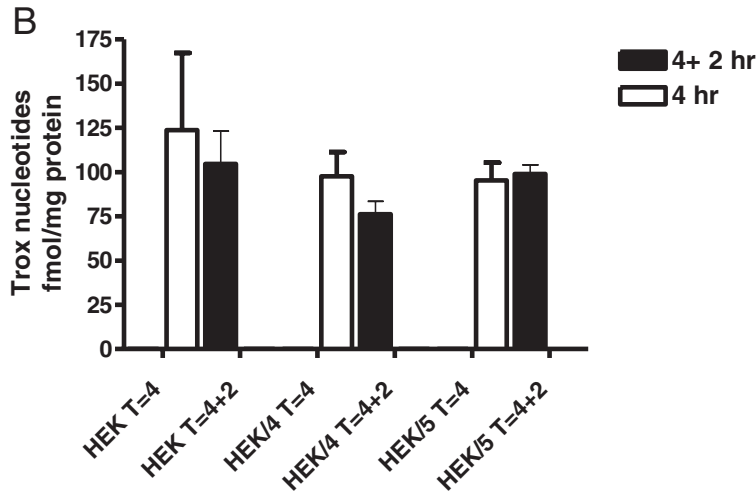

Supplement: Supplementary file 5 — Authors’ original file for figure 5 [file 40064_2014_1514_MOESM5_ESM.pdf]

Ara-C

A

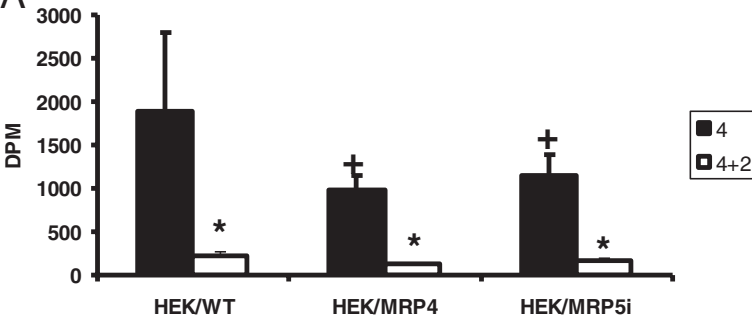

Ara-CxP

B

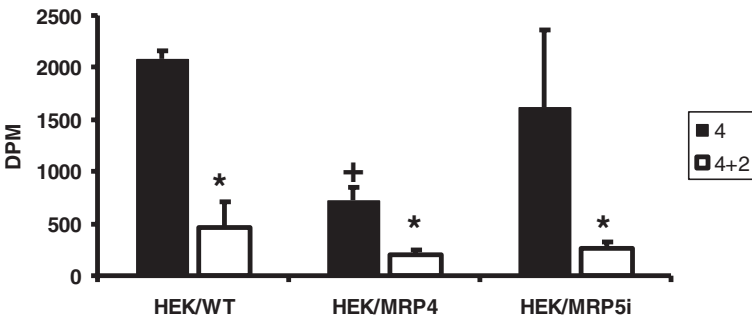

Supplement: Supplementary file 6 — Authors’ original file for figure 6 [file 40064_2014_1514_MOESM6_ESM.pdf]

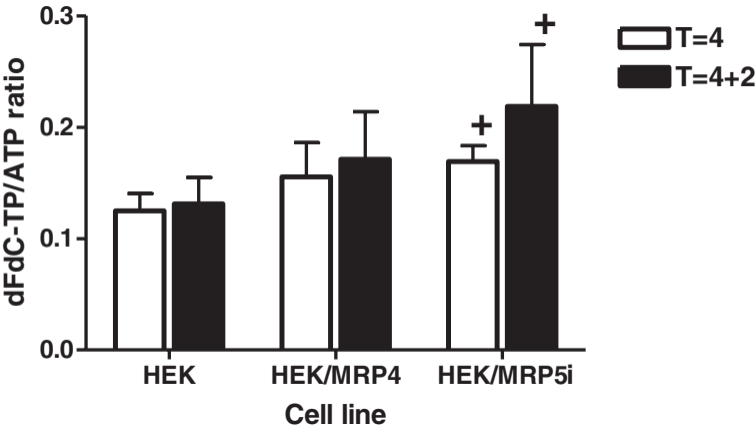

Supplement: Supplementary file 7 — Authors’ original file for figure 7 [file 40064_2014_1514_MOESM7_ESM.pdf]
